# Prevalence of systemic complications of COVID-19 in critically ill patients: systematic review with meta-analysis ^*^

**DOI:** 10.1590/1518-8345.7568.4608

**Published:** 2025-07-11

**Authors:** Namie Okino Sawada, Tiago Marques dos Reis, Patrícia Scotini Freitas, Camila Mendonça de Moraes, Bianca de Moura Peloso-Carvalho, Munyra Rocha Silva Assunção, Camila Maria Silva Paraizo Horvath, Ranile Santos Silva, Karina Dal Sasso Mendes

**Affiliations:** 1 Universidade Federal de Alfenas, Alfenas, MG, Brazil.; 2 Universidade de São Paulo, Escola de Enfermagem de Ribeirão Preto, PAHO/WHO Collaborating Centre for Nursing Research Development, Ribeirão Preto, SP, Brazil.; 3 Universidade Federal do Rio de Janeiro, Escola de Enfermagem, Macaé, RJ, Brazil.; 4 Scholarship holder at the Coordenação de Aperfeiçoamento de Pessoal de Nível Superior (CAPES), Brazil.; 5 Scholarship holder at the Conselho Nacional de Desenvolvimento Científico e Tecnológico (CNPq), Brazil.; 6 Universidade José do Rosário Velano, Faculdade de Medicina, Alfenas, MG, Brazil.

**Keywords:** COVID-19, SARS-CoV-2, Comorbidity, Critical Illness, Critical Care, Systematic Review

## Abstract

to identify the prevalence of systemic complications of COVID-19 in critically ill patients, to estimate the clinical conditions that may have a worse prognosis when associated with COVID-19.

systematic review with meta-analysis of observational studies, based on the recommendations of the JBI Manual for Evidence Synthesis for systematic reviews of prevalence and incidence, in six databases and grey literature, period 2020 to 2024, languages Portuguese, English and Spanish. We identified 2393 publications. The selection, data extraction and methodological evaluation of the studies were carried out by two independent researchers. The meta-analysis was performed using the Freeman Tukey transformational random-effects statistical method.

ten papers were included. The meta-analysis of seven papers dealing with respiratory complications due to COVID-19 demonstrated a mean prevalence of 42%, (95% CI 0.2-0.66) with heterogeneity I^2^=97.4; the meta-analysis of 4 papers involving the prevalence of neurological complications due to COVID-19 showed an average prevalence of 62%, (95% CI 0.49- 0.74) with heterogeneity I^2^=87.4 and the meta-analysis of 8 studies showed a prevalence of mortality due to COVID-19 of 33%, (95% CI 0.23- 0.44) with heterogeneity I^2^=93.6.

respiratory and neurological complications were the most prevalent in the reviewed studies. PROSPERO Registration: CRD42020214617.

## Highlights

**(1)** Respiratory and neurological complications stood out as the most prevalent complication. **(2)** The main risk factors are: being male, presenting comorbidities, having respiratory and cardiac diseases. **(3)** Evidence can guide the practice of health professionals. **(4)** Evidence can assist in the implementation of care for critically ill patients.

## Introduction

The coronavirus, which belongs to the family of the same name and is responsible for the development of respiratory infections, has ravaged the world after the spread of a new strain called SARS-CoV-2, which causes COVID-19. On March 11^th^, 2020, the World Health Organization classified the geographic distribution of the disease as a pandemic, a situation that lasted for almost 30 months^(1-3)^. From then on, preventive measures such as social distancing, quarantine and use of masks were implemented, aiming to reduce the transmissibility and lethality of the disease^(4)^. These measures resulted in psychological suffering and mental disorders, such as anxiety, depression and stress^(5)^. In addition, systemic complications caused by the disease began to be observed, mainly through impairments in the pulmonary and cardiovascular systems^(6-7)^.

On October 14^th^, 2021, the United States Center for Disease Control and Prevention (CDC) showed that COVID-19 did not affect all population groups equally^(8)^. The risk of complications arising from the disease increases proportionally to the number of underlying conditions a person has. Previous studies indicate that individuals with certain disabilities are more likely to contract it and have adverse outcomes^(9-10)^. In addition, some chronic conditions, such as lung disease, heart disease, diabetes, and obesity, are more prevalent in certain minority populations, affecting a younger age group. In the United States of America, an estimated increase of 700,000 excess deaths was observed, with the largest percentage increase in mortality among adults aged 25 to 44 and among Hispanics or Latinos^(8)^. In 2022, COVID-19 was the fourth leading cause of death in that country^(11)^. Evidence supported by systematic reviews highlights that underlying conditions that increase the risk of progression to severe disease due to COVID-19 include lung disease^(12-16)^, cancer^(17-18)^, cerebrovascular disease^(18-21)^, chronic kidney disease^(22-23)^, chronic liver disease (cirrhosis, non-alcoholic fatty liver disease, alcoholic liver disease, autoimmune hepatitis)^(24-26)^, Diabetes Mellitus (DM), Type 1 Diabetes Mellitus (DM1) and Type 2 Diabetes Mellitus (DM2)^(27)^, heart problems (such as heart failure, coronary artery disease or cardiomyopathies)^(28-29)^, current and former smoking^(30-32)^, tuberculosis^(33-34)^, obesity^(35)^, pregnancy and recent pregnancy^(36)^ and mental health disorders (mood disorders, including depression and schizophrenia spectrum disorders)^(37-38)^.

Given the above, risk groups appear to have a higher probability of developing systemic complications from COVID-19. Critical patients were considered to be those who were seriously ill, with clinical instability or risk of instability of the vital system with risk of death and, therefore, admitted to an intensive care unit (ICU)^(39)^. Thus, this study aimed to identify the prevalence of systemic complications of COVID-19 in critically ill patients. It is expected that the study can contribute to estimating the clinical conditions of patients who may have a worse prognosis when associated with COVID-19.

## Method

### Study design

This is a systematic review that followed the guidelines of the Brazilian Center for Evidence-Based Research COBE-UFSC^(40)^ and the Preferred Reporting Items for Systematic Reviews and Meta-Analyses (PRISMA) checklist^(41)^. Six stages were organized for its development: (1) elaboration of the research question; (2) preparation of the preliminary search strategy; (3) selection of databases; (4) definition of eligibility criteria; (5) data collection and analysis of risks of bias; and (6) reporting of the review^(40)^. The systematic review protocol was registered in the International Prospective Register of Ongoing Systematic Reviews – PROSPERO under number CRD42020214617.

### Study question

This review sought to answer the following question: what is the prevalence of systemic complications resulting from SARS-CoV-2 contamination in adults with COVID-19 in critical condition? To formulate the question, PECOS strategy was used^(40)^, as follows: P - Participants: adults with COVID-19; E - Exposure: COVID-19 contamination; C - Control: absence of contamination; O - Outcome: systemic complications; S - Observational studies.

### Data collection period

This systematic review was carried out from August 30^th^, 2020 to March 30^th^, 2024.

### Criteria for eligibility

Observational studies that addressed adults with COVID-19 in critical condition were included, considering epidemiological aspects, clinical manifestations, diagnosis, prevention and control of the disease. As exclusion criteria, review studies, case control studies, case reports, case studies and series, publications of abstracts of annals of scientific events and editorials were disregarded.

### Data collection and search strategy

The databases used were MEDLINE (via PubMed), EMBASE, LILACS, Web of Science Core Collection, Scopus and CINAHL. For the search strategy, the descriptors of Medical Subject Headings (MeSH), Emtree, CINAHL Titles and Health Sciences Descriptors (DeCS/MeSH) were used, in addition to synonymous terms, considering the specificities of each database. As an example, PubMed’s implemented search strategy stands out: ((“COVID-19”[Supplementary Concept] OR “COVID-19”[All Fields] OR “2019 novel coronavirus disease”[All Fields] OR “COVID19”[All Fields] OR “COVID-19 pandemic”[All Fields] OR “SARS-CoV-2 infection$”[All Fields] OR “COVID-19 virus disease”[All Fields] OR “2019 novel coronavirus infection$”[All Fields] OR “2019-nCoV infection$”[All Fields] OR “coronavirus disease 2019”[All Fields] OR “coronavirus disease-19”[All Fields] OR “2019-nCoV disease”[All Fields] OR “COVID-19 virus infection$”[All Fields] OR “Coronavirus Infections”[Mesh] OR “Coronavirus Infections”[All Fields] OR “Coronavirus Infection$”[All Fields] OR “Middle East Respiratory Syndrome”[All Fields] OR “MERS (Middle East Respiratory Syndrome)”[All Fields]) AND (“Systemic complications”[Title/Abstract] OR “Systemic complication$”[All Fields] OR “Covid-19 complication$”[All Fields] OR “Covid 19 complication$”[All Fields] OR “Covid19 complication$”[All Fields] OR “SARS-CoV-2 complication$”[All Fields])). The search was performed on August 30^th^, 2023.

In addition, grey literature was also searched in Google Scholar, Proquest and OpenGrey sources and the reference lists of the included articles were manually searched.

The results identified in each database were exported to EndNote 20 desktop version to remove duplicates. The records were then exported to the online software Rayyan^(42)^ for individual selection of studies by two reviewers, by reading the titles and abstracts, followed by selection of the full text. After each selection phase, a consensus meeting was held to discuss and align conflicts.

### Data collection

Data extraction was conducted using the Joanna Briggs Institute (JBI)^(43)^ collaboration instrument for observational studies, covering the following variables: country, context and location, participant characteristics, groups, outcome measures and description of the main results. Data collection was carried out independently by two researchers and conflicts were resolved with a third reviewer.

### Data analysis

A qualitative synthesis was performed considering dependent variables (type of systemic complication) and independent variables (sex, age group, presence of comorbidities, and laboratory findings). In the quantitative synthesis, a meta-analysis of proportions was performed using the Freeman Tukey transformational random-effects statistical method, with a 95% confidence interval. The meta-analysis was performed using the JBI collaboration software^(43)^, grouping the studies in each group according to similar prevalence in relation to systemic complications of COVID-19. The analyses involved all included articles, regardless of methodological quality. Systemic complications of COVID-19 were classified according to their etiology as: neurological and respiratory or death.

To assess methodological quality, the tool proposed by the JBI collaboration^(44)^ for cohort studies was used, which includes eleven questions: Were the two groups similar and recruited from the same population? Were exposures measured similarly to assign people to the exposed and unexposed groups? Was the exposure measured in a valid and reliable manner? Were confounding factors identified? Were strategies to deal with confounding factors stated? Were the groups/participants free from the outcome at baseline (or at the time of exposure)? Were the outcomes measured in a valid and reliable manner? Was the follow-up time reported and sufficient for the results to occur? Was the follow-up complete and, if not, were the reasons for loss to follow-up described and explored? Were strategies used to address incomplete follow-up? Was adequate statistical analysis used? This assessment was performed by two reviewers independently, and any doubts were discussed in meetings to seek consensus. The overall methodological quality of each study was considered for the interpretation of the analysis, which was classified as “High”, “Moderate” or “Low”, based on the count or percentage of “Yes” responses to the tool items. Thus, we adopted, for the cutoff points, up to 25% of yes responses: “high risk of bias”; from 25 to ≤75%: “moderate” risk of bias; and greater than 75%: “low” risk of bias^(45)^.

The certainty of evidence was not assessed, since there is currently no formal Grading of Recommendations Assessment, Development and Evaluation (GRADE) guidance for systematic reviews of prevalence and incidence^(46)^.

## Results

**Figure 1 -:**
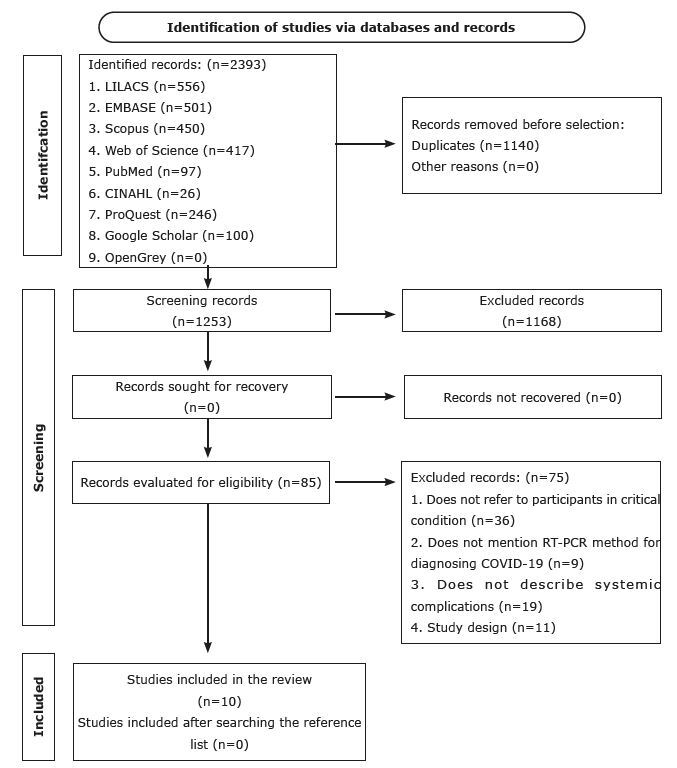
Flowchart of selection of studies included in the systematic review adapted from the Preferred Reporting Items for Systematic Review and Meta-Analyses (PRISMA) (n = 10), 2020-2024

Source: Adapted from PRISMA^(41)^

The search identified 2393 records, 97 from PubMed, 417 from Web of Science Core Collection, 556 from LILACS, 450 from Scopus, 501 from EMBASE, 26 from CINAHL, 100 from Google Scholar and 246 from ProQuest and none from OpenGrey. After removing duplicates and considering the eligibility criteria, 10 cohort studies resulted. Figure 1 shows the flowchart of the selection process of the studies included in the systematic review.

The summary of the 10 studies describes the identification of the study, country of conduct, context, characteristics of the participants, groups, outcome measures and main results and can be viewed at https://doi.org/10.48331/scielodata.4BEZDJ.

**Figure 2 -:**
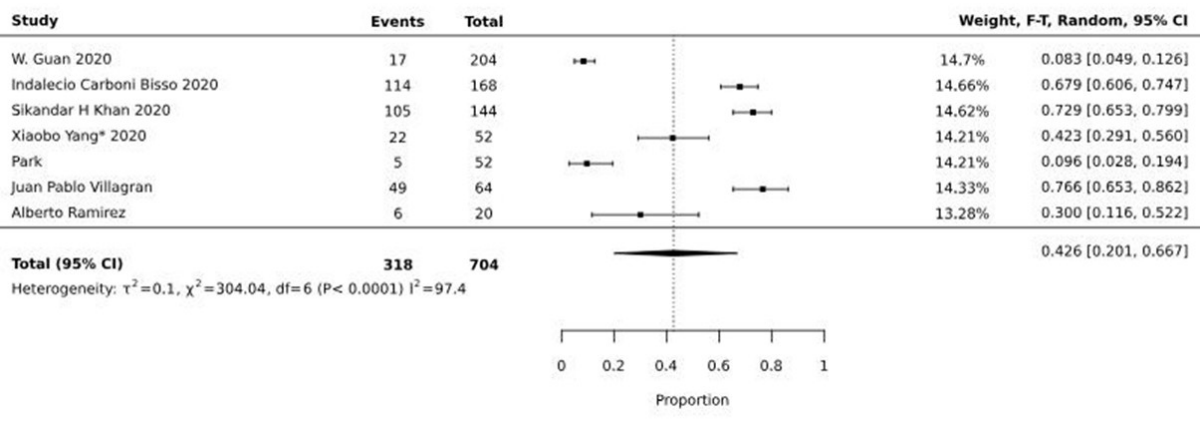
Meta-analysis of seven articles addressing the prevalence of respiratory complications from COVID-19, 2020-2024

**Figure 3 -:**
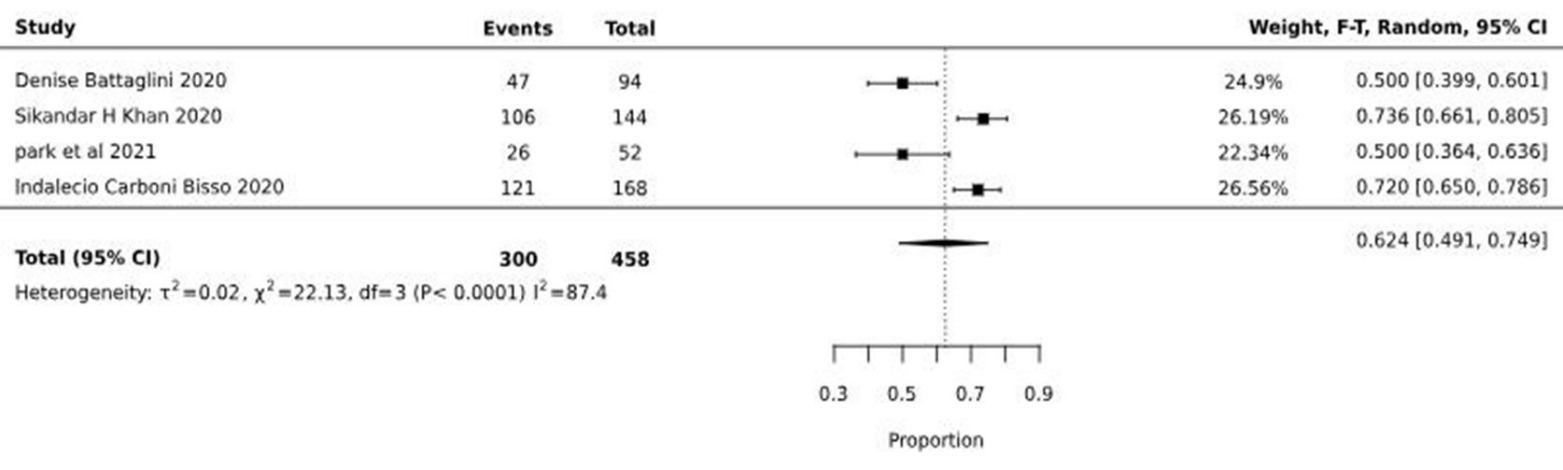
Meta-analysis of 4 studies showing the prevalence of neurological complications from COVID-19, 2020-2024

**Figure 4 -:**
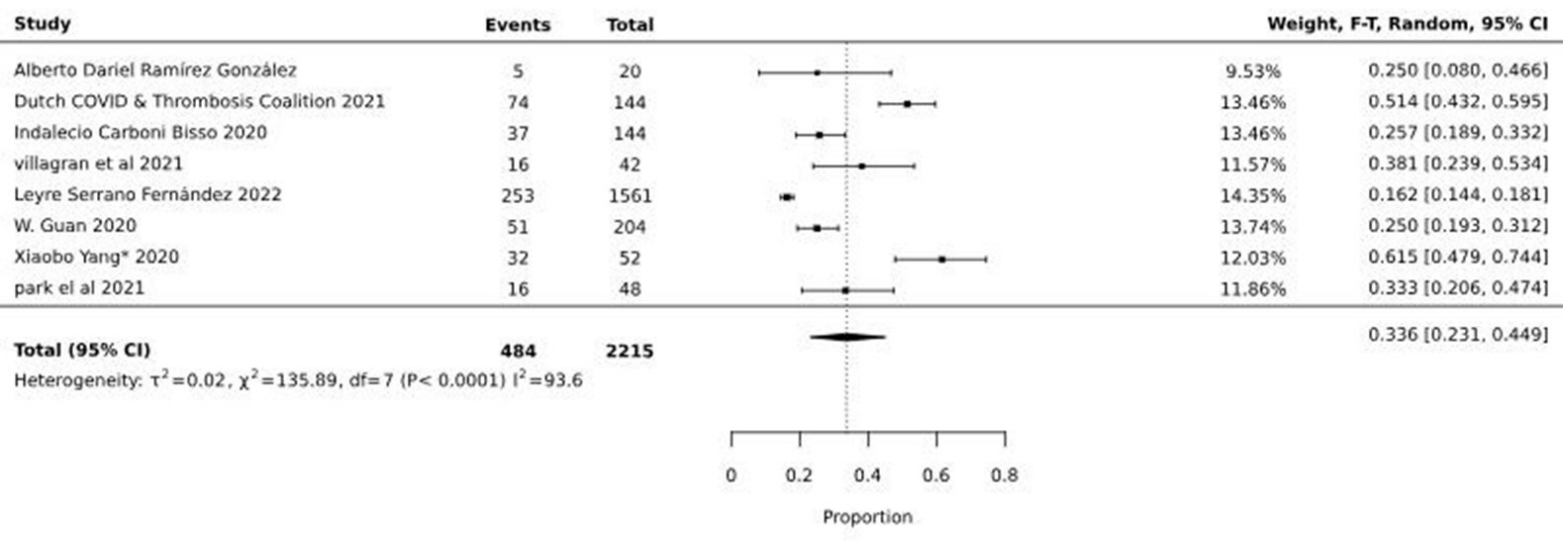
Meta-analysis of eight studies showing the prevalence of COVID-19 mortality, 2020-2024

The proportions of occurrences of systemic complications of COVID-19 are described in Figure 2, Figure 3 and Figure 4 Figures 2, 3 and 4.

Figure 2 shows the meta-analysis of seven articles that dealt with respiratory complications due to COVID-19. It was possible to observe a mean prevalence (meta-prevalence) of 42%, (95%CI 0.2-0.66) with heterogeneity I^2^=97.4.

Figure 3 shows the meta-analysis of four articles that involved the prevalence of neurological complications due to COVID-19. An average prevalence (meta-prevalence) of 62% was observed (95%CI 0.49-0.74) with heterogeneity I^2^=87.4

Figure 4 shows the meta-analysis of eight studies that showed the prevalence of mortality from COVID-19. An average prevalence (meta-prevalence) of 33% can be observed (95%CI 0.23-0.44) with heterogeneity I^2^=93.6.

Heterogeneity was assessed by inconsistency (I²), which describes the percentage variation attributed to them beyond chance. The cutoff points considered were: 25% low; 50% moderate; and 75% high heterogeneity, with a p-value <0.05^(47)^.

**Figure 5 -:**
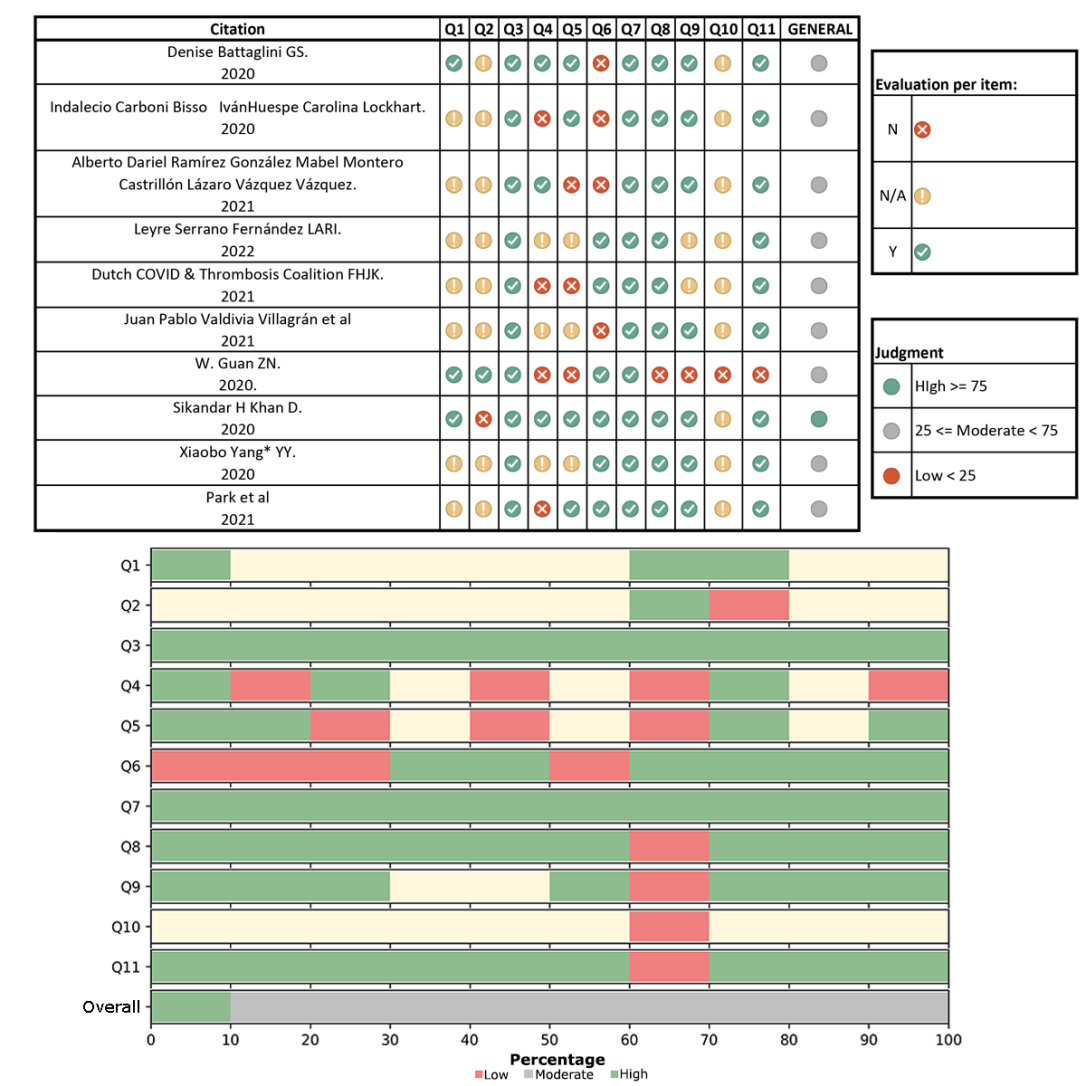
Assessment of methodological quality of included cohort studies (n = 10), 2020-2024

Regarding the assessment of the methodological quality of the included cohort studies, 10% presented high methodological quality and 90% moderate quality, that is, 10% of the studies had a “low” risk of bias and 90% “moderate” risk of bias (Figure 5).

## Discussion

This systematic review asked the question of the prevalence of systemic complications of COVID-19 in critically ill patients. We found that respiratory and neurological complications were the most prevalent in the reviewed studies, indicating that critically ill patients with COVID-19 may be at higher risk of developing these complications. Of the 10 studies included in the systematic review, three were produced in Europe^(48-50)^, three in Asia^(51-53)^, one in Central America^(54)^, one in North America^(55)^ and two in South America^(56-57)^, characterizing COVID-19 as a pandemic with serious repercussions in several countries and regions of the world. The oldest publications date back to 2020, the year the health emergency began^(58)^. The number of participants in the included cohort studies totalled 1,143 people, with an average age of 62 years old, predominantly male. In most studies, patients presented at least one comorbidity. The most prevalent comorbidities were: high blood pressure, diabetes mellitus, obesity, and heart disease.

Regarding pulmonary manifestations in COVID-19, these can manifest as mild, moderate, or severe. In the mild form, symptoms such as body aches, cough, or mild fever may be present. In the moderate form, the disease may present as mild pneumonia, associated with other symptoms. The severe form of the disease may be characterized by severe pneumonia and hypoxia. In critical cases that present significant hypoxia and organ failure, admission to the Intensive Care Unit (ICU) with mechanical ventilatory support may be necessary^(52,59-60)^.

The studies identified in this systematic review revealed that mechanical ventilation was initiated more frequently in patients with severe diseases compared to those with non-severe diseases (non-invasive ventilation, 32.4% versus -vs 0%; invasive ventilation, 14.5% vs 0%)^(51)^. The study^(56)^ involving 168 critically ill patients with COVID-19 (average age 65 years old, 66% were male) indicated that arterial hypertension was the most frequent comorbidity, affecting 52.4% of patients. Of these, 67.9% required Invasive Mechanical Ventilation (IMV), and none were treated with non-invasive ventilation. Most patients on IMV (73.7%) required neuromuscular blockade due to severe hypoxemia and 36% were ventilated in the prone position. The average period for ICU stay was 13 days, with a mortality rate of 25%. The study showed that mechanical ventilation was associated with a higher chance of developing *delirium* (OR: 42.1; 95% CI: 13.0-137.1), with a mortality of 26.4% in patients with *delirium* compared to 15.8% in patients without such disorder^(55)^. Among 710 patients with SARS-CoV-2 pneumonia, 52 critically ill adults were included. The mean age was 59.7 years, 67% were men, and 40% had chronic medical conditions. Most patients (61.5%) died within 28 days, with a median duration from ICU admission to death of 7 days for nonsurvivors. Compared with survivors, nonsurvivors were older, more likely to develop acute respiratory distress syndrome (ARDS) [26 (81%) patients vs 9 (45%) patients], and more likely to receive mechanical ventilation [30 (94%) patients vs 7 (35%) patients], invasive or non-invasive. Most patients had organ dysfunction, including acute respiratory distress syndrome (ARDS) (67%), acute kidney injury (29%), cardiac disease (23%), liver dysfunction (29%), and pneumothorax (2%). Thirty-seven (71%) required IMV, and seven (13.5%) developed hospital infection^(52)^.

This association was also demonstrated in a study^(57)^, in which the most severe cases of COVID-19 had occurred in elderly, hypertensive and obese patients, with a significant increase in mortality when associated with kidney disease.

A study^(54)^ revealed that 30% of patients required IMV, all of whom were aged 65 years or older and predominantly female. Of these, 83.3% died (5), and four had severe or moderate ARDS (RR 16.7) according to the Berlin classification. The mean ideal positive end-expiratory pressure (PEEP) used was 16.5 (SD=2.1) cmH_2_O (CI 15.6-17.4), with the initial ventilatory modality being volume-controlled ventilation (VCV). In one of the cases, the prone position was adopted after the first 12 hours of admission to the ICU. The prone position was contraindicated in other cases due to hemodynamic instability. The patient who was successfully weaned from ventilation used Bi-positive pressure (BiPAP) mode, which resulted in less need for sedation and muscle relaxation, in addition to a shorter time on IMV (5 days), despite the advanced age. Non-invasive ventilation was not used.

The meta-analysis of the prevalence of respiratory complications was 42% (CI: 0.39-0.46, I^2^: 98), resulting in the need for IMV, in a total population of 704 patients admitted to the ICU. The systematic review^(61)^, carried out with the aim of understanding whether the presentations and manifestations of COVID-19 were limited exclusively to pulmonary manifestations, identified an incidence of 33% of Acute Respiratory Syndrome.

Regarding neurological complications, evidence from a systematic review^(62)^ indicated that the most common neurological manifestations associated with COVID-19 included myalgia, headache, sensory alteration, hyposmia, and hypogeusia. In addition, rare central nervous system manifestations, such as ischemic stroke, intracerebral hemorrhage, encephalomyelitis, and acute myelitis, as well as peripheral nerve manifestations, such as Guillain-Barré syndrome and Bell’s palsy, and skeletal muscle manifestations, such as rhabdomyolysis, have also been observed. Recent reports suggest that hypoxic-ischemic damage may be the main cause of neurological symptoms in patients with COVID-19^(63)^.

The studies included in this systematic review identified *delirium* as the most common neurological complication. In one of the studies^(48)^, no association was observed between *delirium* and the duration of mechanical ventilation. On the other hand, another study^(56)^ indicated a lower incidence of this condition in patients not undergoing mechanical ventilation (23, 42.5%) compared to patients on mechanical ventilation (61, 89.7%). One study^(55)^ found that *delirium* occurred in 73.6% of patients and that mechanical ventilation was associated with increased odds of developing *delirium* or coma in 76.4% (110/144). Sixty-three percent of patients tested positive for *delirium* (OR: 42.1, 95% CI: 13.0-137.1).

Another study^(53)^ showed that among 52 ICU patients with COVID-19, the mean age was 73 years old, and 19 (36.5%) had pre-existing neurological comorbidities. New-onset neurological complications occurred in 23 (44.2%) patients during their ICU stay. Those with pre-existing neurological comorbidities required tracheostomy more frequently, as well as more days of ventilation and ICU stay, compared to those without comorbidities.

The meta-analysis of the prevalence of neurological complications, involving 458 patients admitted to the ICU, revealed a high prevalence of 62% (CI: 0.49-0.74, I^2^: 87.4).

The systematic review with meta-analysis^(64)^ highlighted the risk factors for mortality in COVID-19, including a total of 14 studies with 29,909 patients infected by the virus, recording 1,445 cases of death. The study revealed significant associations between advanced age (≥65 vs <65 years) (pooled OR = 4.59, 95% CI = 2.61–8.04, p < 0.001), male gender compared to female gender (pooled OR = 1.50, 95% CIs = 1.06–2.12, p = 0.021), and the risk of death from COVID-19 infection. Additionally, conditions such as hypertension (pooled OR = 2.70, 95% CI = 1.40–5.24, p = 0.003), cardiovascular disease (CVD) (pooled OR = 3.72, 95% CI = 1.77–7.83, p = 0.001), diabetes (pooled OR = 2.41, 95% CI = 1.05–5.51, p = 0.037), chronic obstructive pulmonary disease (COPD) (pooled OR = 3.53, 95% CI = 1.79–6.96, p < 0.001), and cancer (pooled OR = 3.04, 95% CI = 1.80–5.14, p < 0.001) were associated with a higher risk of mortality^(64)^. The study^(49)^ demonstrated a higher percentage of complications during hospitalization in patients with bacteremic pneumococcal pneumonia during hospitalization. In contrast, patients with SARS-CoV-2 pneumonia had a higher in-hospital mortality rate (10.8% vs 6.8%; P=0.004) and longer hospital stay.

In patients with SARS-CoV-2 pneumonia, the predictors of in-hospital mortality were: age ≥65 years (OR 4.777, 95% CI 2.814–8.111; P<0.001); heart disease (OR 1.587, 95% CI 1.007–2.341; P=0.02); liver disease (OR 2.365, 95% CI 1.199–4.662); altered mental status (OR 2.464, 95% CI 1.586–3.827; P<0.001); respiratory rate ≥30 breaths/min (OR 2.117, 95% CI 1.362–3.289; P<0.001); hypoxemia (OR 1.599, 95% CI 1.045–2.447; P=0.03); blood urea nitrogen ≥30 mg/dL (OR 1.864, 95% CI 1.236–2.81; P=0.003); neutrophilia (every 10,000 unit increase in neutrophil count/μL) (OR 1.052, 95% CI 1.018–1.086; P=0.002); bilateral lung involvement (OR 1.868, 95% CI 1.142–3.056; P=0.013); pleural effusion (OR 2.894, 95% CI 1.282–6.536; P=0.011), and septic shock (OR 2.781, 95% CI 1.703–4.542; P<0.001). Seven or more days of symptoms at admission were protective factors against mortality (OR 0.464, 95% CI 0.314–0.685; P<0.001).

The results of this review corroborate the analysis of a study^(64)^, since all included studies highlighted risk factors, with a predominance of males. In seven (70%) of the studies, patients had at least one comorbidity, with a predominance of hypertension, diabetes, obesity, heart and lung diseases. The meta-analysis of mortality prevalence in this systematic review for COVID-19 was 33% (CI: 0.23–0.44, I^2^: 93.6), involving a population of 2215 patients admitted to the ICU.

Another study^(50)^ that evaluated the incidence of thrombotic complications and overall mortality in patients with COVID-19 admitted to the ICU during the first and second waves of the pandemic revealed that, of the 358 patients admitted to the ICU, 144 patients died (15%). The adjusted cumulative incidence of all thrombotic complications after 10, 20, and 30 days was 12% (95% confidence interval (CI) 9.8–15%), 16% (13–19%), and 21% (17–25%), respectively. Patient characteristics between the first and second waves were comparable. The adjusted hazard ratio (HR) for all-cause mortality in the second wave vs the first wave was 0.53 (95% CI 0.41–0.70). The adjusted HR for any thrombotic complication in the second wave vs the first was 0.89 (95% CI 0.65–1.2). In conclusion, a 47% reduction in mortality was observed in the second wave, but the rate of thrombotic complications remained high and comparable to the first wave. The need for cautious care and the guarantee of adequate thromboprophylaxis is emphasized.

Another important aspect is the patient’s admission to the emergency unit. A study^(65)^ identified the importance of using risk classification at the time of admission, in which patients with COVID-19, who had modified early warning scores greater than 4, were associated with the urgent, very urgent and emergency categories and had greater clinical deterioration such as respiratory failure and shock, evolving more often to death. This shows that the risk protocol correctly prioritized patients at risk of death.

As for the limitations of the study, this systematic review did not include texts in Mandarin, as no researchers were found on the team who were fluent in this language. Another limitation was the high heterogeneity of the studies found in the meta-analysis, which we attribute to the set of complications analysed by the papers. Perhaps a knowledge gap would be cohort studies with only one type of complication. In addition, most of the studies presented a moderate risk of bias; only one showed a low risk of bias, so there is a gap in studies with more rigorous methods.

This systematic review provides a significant contribution to the advancement of knowledge about systemic complications in patients admitted to the ICU. By presenting detailed evidence on the prevalence of respiratory and neurological complications and mortality, together with the identification of their risk factors, the review serves as a valuable tool to guide healthcare professionals in planning and providing care to this critical population. The information gathered in this study has the potential to improve clinical practices, promoting a more informed and effective approach to the care of critically ill COVID-19 patients.

## Conclusion

Respiratory and neurological complications stood out as the most prevalent among critically ill patients with COVID-19 in the reviewed studies. Significant risk factors for complications and mortality related to COVID-19, such as male gender and comorbidities such as hypertension, diabetes mellitus, obesity, and respiratory and cardiac diseases, were consistently present in most of the studies analyzed. The findings highlight the importance of early identification and careful management of these risk factors to improve clinical outcomes in critically ill patients with COVID-19.

This review provides evidence that can guide the practice of health professionals, highlighting the main risk factors associated with respiratory and neurological complications in critically ill patients with COVID-19. The results offer valuable support for improving the prevention and management of these conditions, contributing to the design and implementation of more effective care.
