# Supplementary material for: Prevalence of systemic complications of COVID-19 in critically ill patients: systematic review with meta-analysis
Source: Rev Lat Am Enfermagem. 2025 Jul 11;33:e4608. doi: 10.1590/1518-8345.7568.4608 (PMC12259223; doi:10.1590/1518-8345.7568.4608)
Supplement: Translation

# Translation: Prevalência de complicações sistêmicas da COVID-19 em pacientes críticos: revisão sistemática com metanálises ^*^

**DOI:** 10.1590/1518-8345.7568.4609

**Authors:** Namie Okino Sawada, Tiago Marques dos Reis, Patrícia Scotini Freitas, Camila Mendonça de Moraes, Bianca de Moura Peloso-Carvalho, Munyra Rocha Silva Assunção, Camila Maria Silva Paraizo Horvath, Ranile Santos Silva, Karina Dal Sasso Mendes

**Affiliations:** 1 Universidade Federal de Alfenas, Alfenas, MG, Brasil.; 2 Universidade de São Paulo, Escola de Enfermagem de Ribeirão Preto, Centro Colaborador da OPAS/OMS para o Desenvolvimento da Pesquisa em Enfermagem, Ribeirão Preto, SP, Brasil.; 3 Universidade Federal do Rio de Janeiro, Escola de Enfermagem, Macaé, RJ, Brasil.; 4 Bolsista da Coordenação de Aperfeiçoamento de Pessoal de Nível Superior (CAPES), Brasil.; 5 Bolsista do Conselho Nacional de Desenvolvimento Científico e Tecnológico (CNPq), Brasil.; 6 Universidade José do Rosário Velano, Faculdade de Medicina, Alfenas, MG, Brasil.

**Keywords:** COVID-19, SARS-CoV-2, Comorbidade, Estado Terminal, Cuidados Críticos, Revisão Sistemática

## Abstract

identificar a prevalência das complicações sistêmicas da COVID-19 em pacientes críticos, para estimar as condições clínicas que podem apresentar pior prognóstico quando associados à COVID-19.

revisão sistemática com metanálise de estudos observacionais, baseada nas recomendações do JBI *Manual for Evidence Synthesis* para revisões sistemáticas de prevalência e incidência, em seis bases de dados e literatura cinzenta, período 2020 a 2024, idiomas português, inglês e espanhol, tendo sido identificadas 2393 publicações. A seleção, a extração dos dados e a avaliação metodológica dos estudos foram realizadas por dois pesquisadores independentes. A metanálise foi realizada pelo método estatístico de Freeman Tukey transformacional de efeito randômico.

dez artigos foram incluídos. A metanálise de sete artigos que tratavam das complicações respiratórias por COVID-19, a prevalência média foi de 42%, (IC95% 0,2- 0,66) com heterogeneidade I^2^=97,4; a metanálise de quatro artigos que envolveram as complicações neurológicas por COVID-19 mostrou prevalência média de 62%, (IC95% 0,49- 0,74) com heterogeneidade I^2^=87,4 e a metanálise de oito estudos mostrou uma prevalência da mortalidade por COVID-19 de 33%, (IC95% 0,23- 0,44) com heterogeneidade I^2^=93,6.

as complicações respiratórias e neurológicas foram as mais prevalentes nos estudos revisados. Registro PROSPERO: CRD42020214617

## Destaques

**(1)** As complicações respiratórias e neurológicas destacaram-se como as mais prevalentes. **(2)** Principais fatores de risco são sexo masculino, comorbidades, doenças respiratórias e cardíacas. **(3)** Evidências podem orientar a práxis dos profissionais de saúde. **(4)** Evidências podem auxiliar na implementação de assistência aos pacientes críticos.

## Introdução

O coronavírus, pertencente à família homônima e responsável pelo desenvolvimento de infecções respiratórias, assolou o mundo após a disseminação de uma nova cepa denominada SARS-CoV-2, causadora da doença COVID-19. Em 11 de março de 2020, a Organização Mundial da Saúde classificou a distribuição geográfica da doença como pandemia, situação que perdurou por quase 30 meses^(1-3)^. A partir de então, foram implementadas medidas de prevenção como distanciamento social, quarentena e uso de máscaras, visando reduzir a transmissibilidade e a letalidade da doença^(4)^. Essas medidas resultaram em sofrimento psicológico e transtornos mentais, como ansiedade, depressão e estresse^(5)^. Além disso, complicações sistêmicas causadas pela doença passaram a ser observadas, principalmente por meio de comprometimentos nos sistemas pulmonar e cardiovascular^(6-7)^.

O Centro de Controle e Prevenção de Doenças dos Estados Unidos da América (CDC), em 14 de outubro de 2021, mostrou que a COVID-19 não afetou de maneira equitativa todos os grupos populacionais^(8)^. O risco de complicações decorrentes da doença aumenta proporcionalmente ao número de condições subjacentes que apresenta uma pessoa. Estudos prévios indicam que indivíduos com certas deficiências têm maior probabilidade de contraí-la e apresentar desfechos adversos^(9-10)^. Além disso, algumas condições crônicas, como doenças pulmonares, doenças cardíacas, diabetes, obesidade, são mais prevalentes em determinadas populações minoritárias, afetando uma faixa etária mais jovem. Nos Estados Unidos da América, observou-se um aumento estimado de 700.000 mortes em excesso, com o maior incremento percentual na mortalidade entre adultos de 25 a 44 anos e entre hispânicos ou latinos^(8)^. Em 2022, a COVID-19 foi a quarta principal morte naquele país^(11)^.

Evidências respaldadas por revisões sistemáticas destacam que condições subjacentes que aumentam o risco de progressão para doença grave devido à COVID-19 incluem doenças pulmonares^(12-16)^, câncer^(17-18)^, doença cerebrovascular^(18-21)^, doença renal crônica^(22-23)^, doença hepática crônica (cirrose, doença hepática gordurosa não alcoólica, doença hepática alcoólica, hepatite autoimune)^(24-26)^, Diabetes Mellitus (DM), Diabetes Mellitus Tipo 1 (DM1) e Diabetes Mellitus Tipo 2 (DM2)^(27)^, problemas cardíacos (como insuficiência cardíaca, doença arterial coronariana ou cardiomiopatias)^(28-29)^, tabagismo atual e antigo^(30-32)^, tuberculose^(33-34)^, obesidade^(35)^, gravidez e gravidez recente^(36)^ e transtornos de saúde mental (transtornos do humor, incluindo depressão e transtornos do espectro da esquizofrenia)^(37-38)^.

Diante do exposto, os grupos de risco parecem ter uma probabilidade maior de desenvolver complicações sistêmicas pela COVID-19. Considerou-se como pacientes críticos aqueles gravemente doentes, com instabilidade clínica ou risco de instabilidade de sistema vital com risco de morte e, dessa forma, internados em unidade de terapia intensiva (UTI)^(39)^. Assim, este estudo teve como objetivo identificar a prevalência das complicações sistêmicas da COVID-19 em pacientes críticos. Espera-se que o estudo possa contribuir para estimar as condições clínicas dos pacientes que podem apresentar pior prognóstico quando associados à COVID-19.

## Método

### Tipo de estudo

Trata-se de uma revisão sistemática que seguiu as diretrizes do Centro Brasileiro de Pesquisas Baseadas em Evidências COBE-UFSC^(40)^, e o *checklist Preferred Reporting Items for Systematic Reviews and Meta-Analyses* (PRISMA)^(41)^. Para o seu desenvolvimento, foram organizadas seis etapas: (1) elaboração da pergunta de pesquisa; (2) preparo da estratégia de busca preliminar; (3) escolha das bases de dados; (4) definição dos critérios de elegibilidade; (5) coleta de dados e análise dos riscos de viés e (6) relato da revisão^(40)^. O protocolo da revisão sistemática foi registrado no *International Prospective Register of Ongoing Systematic Reviews* – PROSPERO com o número CRD42020214617.

### Pergunta de estudo

Buscou-se, com esta revisão, responder à seguinte pergunta: qual a prevalência das complicações sistêmicas resultantes da contaminação pelo SARS-CoV-2 em adultos com COVID-19 em estado crítico? Para formular a pergunta, utilizou-se da estratégia PECOS^(40)^, sendo: P - Participantes: adultos com COVID-19; E - Exposição: contaminação por COVID-19; C - Controle: ausência de contaminação; O - Desfecho: complicações sistêmicas; S - estudos observacionais.

### Período de coleta de dados

A realização dessa revisão sistemática compreendeu o período de 30 de agosto de 2020 a 30 de março de 2024.

### Critérios de elegibilidade

Foram incluídos estudos observacionais que abordaram adultos com COVID-19 em condição crítica, considerando aspectos epidemiológicos, manifestações clínicas, diagnóstico, prevenção e controle da doença. Como critério de exclusão, foram desconsiderados estudos de revisão, estudos de caso controle, relatos de caso, estudos e séries de caso, publicações de resumos de anais de eventos científicos e editoriais.

### Levantamento dos dados e estratégia de busca

As bases de dados utilizadas foram MEDLINE (via *PubMed*), EMBASE, LILACS, *Web of Science Core Collection*, *Scopus* e CINAHL. Para a estratégia de busca, foram empregados os descritores do *Medical Subject Headings* (MeSH), Emtree, Títulos CINAHL e os Descritores em Ciências da Saúde (DeCS/MeSH), além dos termos sinônimos, considerando as especificidades de cada base de dados. Como exemplo, destaca-se a estratégia de busca implementada na *PubMed*: ((“COVID-19”[Supplementary Concept] OR “COVID-19”[All Fields] OR “2019 novel coronavirus disease”[All Fields] OR “COVID19”[All Fields] OR “COVID-19 pandemic”[All Fields] OR “SARS-CoV-2 infection$”[All Fields] OR “COVID-19 virus disease”[All Fields] OR “2019 novel coronavirus infection$”[All Fields] OR “2019-nCoV infection$”[All Fields] OR “coronavirus disease 2019”[All Fields] OR “coronavirus disease-19”[All Fields] OR “2019-nCoV disease”[All Fields] OR “COVID-19 virus infection$”[All Fields] OR “Coronavirus Infections”[Mesh] OR “Coronavirus Infections”[All Fields] OR “Coronavirus Infection$”[All Fields] OR “Middle East Respiratory Syndrome”[All Fields] OR “MERS (Middle East Respiratory Syndrome)”[All Fields]) AND (“Systemic complications”[Title/Abstract] OR “Systemic complication$”[All Fields] OR “Covid-19 complication$”[All Fields] OR “Covid 19 complication$”[All Fields] OR “Covid19 complication$”[All Fields] OR “SARS-CoV-2 complication$”[All Fields])). A busca foi realizada em 30 de agosto de 2023.

Em adição, foi investigada também a literatura cinzenta, nas fontes *Google Scholar, Proquest* e *OpenGrey* e a busca manual na lista de referências dos artigos incluídos.

Os resultados identificados em cada base de dados foram exportados para o *EndNote* 20 versão *desktop* para remoção das duplicações. A seguir, os registros foram exportados para o *software onlineRayyan* ^(42)^, para a seleção dos estudos de forma individualizada por dois revisores, por meio da leitura dos títulos e resumos, seguida da seleção do texto na íntegra. Após cada uma das fases de seleção, realizou-se uma reunião de consenso para discussão e alinhamento dos conflitos.

### Coleta de dados

A extração dos dados foi conduzida utilizando o instrumento da colaboração *Joanna Briggs Institute* (JBI)^(43)^ para estudos observacionais, contemplando as seguintes variáveis: país, contexto e local, características dos participantes, grupos, medidas de resultados e descrição dos principais resultados. A coleta foi realizada por dois pesquisadores de forma independente e os conflitos resolvidos com um terceiro revisor.

### Análise dos dados

Realizou-se uma síntese qualitativa considerando variáveis dependentes (tipo de complicação sistêmica) e independentes (sexo, faixa etária, presença de comorbidades e achados laboratoriais). Na síntese quantitativa, procedeu-se a uma metanálise de proporção utilizando o método estatístico de Freeman Tukey transformacional de efeito randômico, com intervalo de confiança de 95%. A metanálise foi realizada por meio do *software* da colaboração JBI^(43)^, agrupando os estudos em cada grupo de acordo com prevalências semelhantes em relação às complicações sistêmicas da COVID-19. As análises envolveram todos os artigos incluídos, independentemente da qualidade metodológica. As complicações sistêmicas da COVID-19 foram classificadas segundo sua etiologia em: neurológicas e respiratórias ou morte.

Para avaliar a qualidade metodológica, utilizou-se a ferramenta proposta pela colaboração JBI^(44)^ para estudos de coorte, que abrange onze questões: os dois grupos eram semelhantes e recrutados na mesma população? As exposições foram medidas de forma semelhante para atribuir as pessoas aos grupos expostos e não expostos? A exposição foi medida de forma válida e confiável? Foram identificados fatores de confusão? Foram declaradas estratégias para lidar com fatores de confusão? Os grupos/participantes estavam livres do desfecho no início do estudo (ou no momento da exposição)? Os resultados foram medidos de forma válida e confiável? O tempo de acompanhamento foi relatado e suficiente para que os resultados ocorressem? O acompanhamento foi completo e, caso contrário, os motivos da perda do acompanhamento foram descritos e explorados? Foram utilizadas estratégias para abordar o acompanhamento incompleto? Foi utilizada análise estatística adequada? Essa avaliação foi realizada por dois revisores de forma independente, sendo as dúvidas discutidas em reuniões para buscar consenso. Para a interpretação da análise foram consideradas a qualidade metodológica geral de cada estudo que é classificada como “Alta”, “Moderada” ou “Baixa”, a partir da contagem ou porcentagem de respostas “Sim” para os itens da ferramenta. Dessa forma, adotamos, para os pontos de corte, até 25% de resposta sim: “alto risco de viés”; de 25 a ≤75%: “moderado” risco de viés; e maior que 75%: “baixo” risco de viés^(45)^.

A certeza da evidência não foi realizada, uma vez que atualmente não existe orientação formal do *Grading of Recommendations Assessment, Development and Evaluation* (GRADE) para revisões sistemáticas de prevalência e incidência^(46)^.

## Resultados

A busca identificou 2393 registros, sendo 97 da *PubMed*, 417 da *Web of Science Core Collection*, 556 da LILACS, 450 da *Scopus*, 501 da EMBASE, 26 da Cinahl, 100 do *Google Scholar*, 246 da *ProQuest* e nenhum da *OpenGrey*. Após a remoção das duplicatas e considerando os critérios de elegibilidade, resultaram 10 estudos de coorte. A Figura 1 mostra o fluxograma do processo de seleção dos estudos incluídos na revisão sistemática.

**Figura 1 -:** Fluxograma de seleção dos estudos incluídos na revisão sistemática adaptado do *Preferred Reporting Items for Systematic Review and Meta-Analyses* (PRISMA) (n = 10), 2020-2024

Fonte: Adaptado do PRISMA^(41)^

A síntese dos 10 estudos descreve a identificação do estudo, país de realização, contexto, características dos participantes, grupos, medidas de resultados e principais resultados e podem ser visualizados em https://doi.org/10.48331/scielodata.4BEZDJ.

As proporções de ocorrências das complicações sistêmicas da COVID-19 estão descritas nas Figuras 2, 3 e 4.

A Figura 2 mostra a metanálise de sete artigos que tratavam das complicações respiratórias por COVID-19. Foi possível observar uma prevalência média (*meta-prevalence*) de 42%, (IC95% 0,2- 0,66) com heterogeneidade I^2^=97,4.

**Figura 2 -:** Metanálise de sete artigos que tratavam da prevalência de complicações respiratórias por COVID-19, 2020-2024

A Figura 3 mostra a metanálise de quatro artigos que envolveram a prevalência das complicações neurológicas por COVID-19. Observa-se uma prevalência média (*meta-prevalence*) de 62%, (IC95% 0,49- 0,74) com heterogeneidade I^2^=87,4

**Figura 3 -:** Metanálise de quatro estudos que mostraram a prevalência de complicações neurológicas por COVID-19, 2020-2024

A Figura 4 evidencia a metanálise de oito estudos que mostraram a prevalência da mortalidade por COVID-19. Pode-se observar uma prevalência média (*meta-prevalence*) de 33%, (IC95% 0,23- 0,44) com heterogeneidade I^2^=93,6.

**Figura 4 -:** Metanálise de oito estudos que mostraram a prevalência da mortalidade por COVID-19, 2020-2024

A heterogeneidade foi avaliada pela inconsistência (I²) que descreve a variação percentual a eles atribuída além do acaso. Os pontos de corte considerados foram: 25% baixa; 50% moderada, e 75% alta heterogeneidade, com valor de p<0,05^(47)^.

Com relação à avaliação da qualidade metodológica dos estudos de coorte incluídos, 10% apresentaram qualidade metodológica alta e 90% qualidade moderada, ou seja 10% dos estudos tem “baixo” risco de viés e 90% “moderado” risco de viés (Figura 5).

**Figura 5 -:** Avaliação da qualidade metodológica dos estudos de coorte incluídos (n = 10), 2020-2024

## Discussão

Esta revisão sistemática teve como pergunta qual a prevalência de complicações sistêmicas da COVID-19 em pacientes críticos e, dessa forma, encontrou-se como resposta que as complicações respiratórias e neurológicas foram as mais prevalentes nos estudos revisados, indicando que pacientes críticos com COVID-19 podem apresentar maior risco de desenvolver essas complicações.

Dos 10 estudos incluídos na revisão sistemática, três foram produzidos na Europa^(48-50)^, três na Ásia^(51-53)^, um na América Central^(54)^, um na América do Norte^(55)^ e dois na América do Sul^(56-57)^, caracterizando a COVID-19 como uma pandemia com reflexos de gravidade em vários países e regiões do mundo.

As publicações mais antigas datam de 2020, ano de início da emergência sanitária^(58)^. O número de participantes dos estudos de coorte incluídos totalizou 1143 pessoas, com média de 62 anos, predominantemente do sexo masculino. Na maioria dos estudos, os pacientes apresentaram pelo menos uma comorbidade, sendo as mais prevalentes a hipertensão arterial, DM, obesidade e doenças cardíacas.

Com relação às manifestações pulmonares na COVID-19, estas podem manifestar-se de forma leve, moderada ou grave. Na forma leve, sintomas como dores no corpo, tosse ou febre leve podem estar presentes. Na forma moderada, a doença pode se apresentar como uma pneumonia leve, associada aos outros sintomas. Já a forma grave da doença pode ser caracterizada por pneumonia grave e hipóxia. Em casos críticos que apresentam hipóxia significativa e falência de órgãos, pode ser necessária internação em Unidade de Terapia Intensiva (UTI) com suporte ventilatório mecânico^(52,59-60)^.

Os estudos identificados nesta revisão sistemática revelaram que a ventilação mecânica foi iniciada com maior frequência em pacientes com doenças graves em comparação com aqueles com doenças não graves (ventilação não invasiva, 32,4% *versus* -vs 0%; ventilação invasiva, 14,5% vs 0%)^(51)^. Um estudo que envolveu 168 pacientes gravemente doentes com COVID-19 (média de idade de 65 anos, 66% do sexo masculino), indicou que a hipertensão arterial foi a comorbidade mais frequente, afetando 52,4% dos pacientes^(56)^. Desses, 67,9% necessitaram de Ventilação Mecânica Invasiva (VMI), e nenhum foi tratado com ventilação não invasiva. A maioria dos pacientes sob VMI (73,7%) precisou de bloqueio neuromuscular devido à hipoxemia grave e 36% foram ventilados em posição prona. A permanência média na UTI foi de 13 dias, com uma taxa de mortalidade de 25%. O estudo evidenciou que a ventilação mecânica estava associada com as maiores chances de desenvolver *delirium* (OR: 42,1; IC95%: 13,0-137,1), com uma mortalidade de 26,4% nos pacientes com *delirium* em comparação com 15,8% nos pacientes sem tal distúrbio^(55)^.

Entre 710 pacientes com pneumonia por SARS-CoV-2, 52 adultos gravemente doentes foram incluídos. A média de idade foi de 59,7 anos, sendo 67% homens e 40% com doenças crônicas. A maioria dos pacientes (61,5%) faleceu em 28 dias, com mediana de 7 dias desde a admissão na UTI até a morte para os não sobreviventes. Comparados aos sobreviventes, os não sobreviventes eram mais velhos, tinham maior probabilidade de desenvolver Síndrome Respiratória Aguda [26 (81%) pacientes vs 9 (45%) pacientes] e maior probabilidade de receber ventilação mecânica [30 (94%) pacientes vs 7 (35%) pacientes], invasiva ou não invasiva. A maioria dos pacientes apresentava disfunção orgânica, incluindo Síndrome do Desconforto Respiratório Agudo (SDRA) (67%), lesão renal aguda (29%), doença cardíaca (23%), disfunção hepática (29%) e pneumotórax (2%). Trinta e sete (71%) necessitaram de VMI, e sete (13,5%) desenvolveram infecção hospitalar^(52)^.

Essa associação também foi evidenciada em um estudo em que os casos mais graves de COVID-19 ocorrem em pacientes idosos, hipertensos e obesos, com um aumento significativo da mortalidade quando associados à doença renal^(57)^.

Outro estudo mostrou que 30% dos pacientes necessitaram de VMI, sendo todos com idade igual ou superior a 65 anos e predominantemente do sexo feminino^(54)^. Destes, 83,3% faleceram (5), sendo que quatro apresentaram SDRA grave ou moderada (RR 16,7) de acordo com a classificação de Berlim. A média de pressão positiva expiratória final ideal (PEEP) utilizada foi de 16,5 (DP=2,1) cmH_2_O (IC 15,6-17,4), com a modalidade ventilatória inicial sendo ventilação controlada por volume (VCV). Em um dos casos, adotou-se a posição em decúbito ventral após as primeiras 12 horas de admissão na UTI. A posição prona foi contraindicada nos demais casos devido à instabilidade hemodinâmica. O paciente que obteve sucesso no desmame ventilatório utilizou o modo Bipressão positiva (BiPAP), o que resultou em menores necessidades de sedação e relaxamento muscular, além de um tempo menor de VMI (5 dias), apesar da idade avançada. Não foi empregada ventilação não invasiva.

A metanálise de prevalência das complicações respiratórias foi de 42% (IC:0,39-0,46, I^2^:98), resultando na necessidade de VMI, em uma população total de 704 pacientes internados em UTI. A revisão sistemática^(61)^, elaborada com objetivo de entender se as apresentações e manifestações da COVID-19 limitaram-se, exclusivamente, às manifestações pulmonares, identificou a incidência de 33% de Síndrome Respiratória Aguda.

Com relação às complicações neurológicas, as evidências provenientes de uma revisão sistemática^(62)^ indicaram que as manifestações neurológicas mais comuns associadas à COVID-19 incluíram mialgia, cefaleia, alteração sensorial, hiposmia e hipogeusia. Além disso, manifestações raras do sistema nervoso central, como acidente vascular cerebral isquêmico, hemorragia intracerebral, encefalomielite e mielite aguda, bem como manifestações nervosas periféricas, como síndrome de Guillain-Barré e paralisia de Bell, e manifestações musculares esqueléticas, como rabdomiólise, também foram observadas. Relatórios recentes sugerem que o dano hipóxico-isquêmico pode ser a principal causa dos sintomas neurológicos em pacientes com COVID-19^(63)^.

Os estudos incluídos nesta revisão sistemática identificaram o *delirium* como a complicação neurológica mais comum. Em um dos estudos^(48)^ não foi observada associação entre o *delirium* e a duração da ventilação mecânica. Por outro lado, outro trabalho^(56)^ indicou uma menor incidência desse agravo em pacientes não submetidos à ventilação mecânica (23, 42,5%) em comparação aos pacientes em ventilação mecânica (61, 89,7%). Uma investigação^(55)^ revelou que o *delirium* ocorreu em 73,6% dos pacientes e que a ventilação mecânica esteve associada às maiores chances de desenvolvimento de *delirium* ou coma em 76,4% (110/144). Sessenta e três por cento dos pacientes foram positivos para *delirium* (OR: 42,1, IC95%: 13,0-137,1).

Outra pesquisa^(53)^ mostrou que, entre 52 pacientes de UTI com COVID-19, a média de idade foi de 73 anos, sendo que 19 (36,5%) tinham comorbidades neurológicas preexistentes. Complicações neurológicas de início recente ocorreram em 23 (44,2%) pacientes durante a internação na UTI. Aqueles com comorbidades neurológicas preexistentes necessitaram de traqueostomia com mais frequência, além de mais dias de ventilação e permanência na UTI, em comparação com os sem comorbidades.

A metanálise da prevalência de complicações neurológicas, envolvendo 458 pacientes internados em UTI, revelou uma prevalência elevada de 62% (IC:0,49-0,74, I^2^:87,4).

A revisão sistemática com metanálise^(64)^ evidenciou os fatores de risco para mortalidade na COVID-19, incluindo um total de 14 estudos com 29.909 pacientes infectados pelo vírus, registrando 1.445 casos de morte. O estudo revelou associações significativas entre idade avançada (≥65 vs <65 anos) (OR agrupadas = 4,59, IC95% = 2,61–8,04, *p*< 0,001), sexo masculino em comparação ao feminino (OR agrupadas = 1,50, 95% ICs = 1,06–2,12, *p*= 0,021), e o risco de morte por infecção por COVID-19.

Adicionalmente, condições como hipertensão (OR agrupada = 2,70, IC95% = 1,40–5,24, *p*= 0,003), doenças cardiovasculares (DCV) (OR agrupada = 3,72, IC 95% = 1,77–7,83, *p*= 0,001), DM (OR agrupadas = 2,41, IC95% = 1,05–5,51, *p*= 0,037), doença pulmonar obstrutiva crônica (DPOC) (OR agrupadas = 3,53, IC95% = 1,79–6,96, *p*< 0,001), e câncer (OR agrupados = 3,04, IC95% = 1,80–5,14, *p*< 0,001) mostraram-se associadas a um maior risco de mortalidade^(64)^.

Já a pesquisa^(49)^ mostrou uma porcentagem maior de complicações durante a internação de pacientes com pneumonia pneumocócica bacteriêmica durante a internação. Em contraste, os pacientes com pneumonia por SARS-CoV-2 apresentaram maior taxa de mortalidade hospitalar (10,8% vs 6,8%; P=0,004) e maior tempo de internação hospitalar.

Em pacientes com pneumonia por SARS-CoV-2, os preditores de mortalidade hospitalar foram: idade ≥65 anos (OR 4,777, IC95% 2,814–8,111; P<0,001); doença cardíaca (OR 1,587, IC95% 1,007–2,341; P=0,02); doença hepática (OR 2,365, IC95% 1,199–4,662); estado mental alterado (OR 2,464, IC95% 1,586–3,827; P<0,001); frequência respiratória ≥30 respirações/min (OR 2,117, IC95% 1,362–3,289; P<0,001); hipoxemia (OR 1,599, IC95% 1,045–2,447; P=0,03); nitrogênio ureico no sangue ≥30 mg/dL (OR 1,864, IC95% 1,236–2,81; P=0,003); neutrofilia (cada aumento de 10.000 unidades na contagem de neutrófilos/μL) (OR 1,052, IC95% 1,018–1,086; P=0,002); envolvimento pulmonar bilateral (OR 1,868, IC95% 1,142–3,056; P=0,013); derrame pleural (OR 2,894, IC95% 1,282–6,536; P=0,011), e choque séptico (OR 2,781, IC95% 1,703–4,542; P<0,001). Sete dias ou mais de sintomas na admissão foram fatores de proteção contra mortalidade (OR 0,464, IC95% 0,314–0,685; P<0,001.

Os resultados desta revisão corroboram com a análise de estudo^(64)^, uma vez que todos os incluídos destacaram fatores de risco, com predomínio do sexo masculino. Em sete (70%) das pesquisas, os pacientes apresentaram pelo menos uma comorbidade, com predominância da hipertensão, DM, obesidade, doenças cardíacas e pulmonares. A metanálise de prevalência de mortalidade nesta revisão sistemática para COVID-19 foi de 33% (IC:0,23-0,44, I^2^:93,6), envolvendo uma população de 2215 pacientes internados em UTI.

Outro estudo^(50)^ que avaliou a incidência de complicações trombóticas e a mortalidade geral em pacientes com COVID-19 internados em UTI durante a primeira e segunda onda da pandemia, revelou que, dos 358 pacientes internados em UTI, 144 pacientes faleceram (15%). A incidência cumulativa ajustada de todas as complicações trombóticas após 10, 20 e 30 dias foi de 12% (intervalo de confiança (IC) de 95% 9,8–15%), 16% (13–19%) e 21% (17–25%), respectivamente. As características dos pacientes entre a primeira e a segunda onda foram comparáveis. A taxa de risco ajustada (HR) para mortalidade geral na segunda onda vs a primeira onda foi de 0,53 (IC95% 0,41–0,70). O HR ajustado para qualquer complicação trombótica na segunda onda vs. a primeira foi de 0,89 (IC95% 0,65–1,2). Em conclusão, observou-se uma redução de 47% na mortalidade na segunda onda, mas a taxa de complicações trombóticas permaneceu elevada e comparável à primeira onda. Ressalta-se a necessidade de cuidados cautelosos e a garantia de uma tromboprofilaxia adequada.

Um outro aspecto importante é a admissão do paciente na unidade de emergência. Um estudo^(65)^ identificou a importância do uso da classificação de risco no momento da admissão, em que pacientes com COVID-19, que tiveram pontuações de alerta precoce modificadas maiores que 4, estavam associados às categorias urgente, muito urgente e emergencial e tiveram maior deterioração clinica como insuficiência respiratória e choque, evoluindo mais para óbito. Isso mostra que o protocolo de risco priorizou corretamente os pacientes com risco de morte.

Quanto às limitações do estudo, esta revisão sistemática não incluiu textos no idioma mandarim, pois não foram encontrados na equipe pesquisadores que dominam o idioma. Outra limitação foi a heterogeneidade alta dos estudos encontrados na metanálise, a qual atribuímos ao conjunto de complicações analisadas pelos artigos. Talvez uma lacuna do conhecimento seria estudos de coorte com apenas um tipo de complicação. Além disso, a maioria dos estudos apresentaram moderado risco de viés; apenas um mostrou baixo risco de viés, portanto existe uma lacuna de estudos com métodos mais rigorosos.

Esta revisão sistemática proporciona uma contribuição significativa para o avanço do conhecimento sobre as complicações sistêmicas em pacientes internados em UTI. Ao apresentar evidências detalhadas sobre a prevalência de complicações respiratórias, neurológicas e mortalidade, juntamente com a identificação de seus fatores de risco, a revisão serve como um instrumento valioso para orientar os profissionais de saúde no planejamento e na prestação de assistência a essa população crítica. As informações reunidas neste estudo têm o potencial de aprimorar as práticas clínicas, promovendo uma abordagem mais informada e eficaz no cuidado aos pacientes com COVID-19 em estado crítico.

## Conclusão

As complicações respiratórias e neurológicas destacaram-se como as mais prevalentes entre pacientes críticos acometidos pela COVID-19 nos estudos revisados. Os fatores de risco significativos para complicações e mortalidade relacionadas à COVID-19, como sexo masculino e as comorbidades hipertensão, DM, obesidade, além de doenças respiratórias e cardíacas, estiveram consistentemente presentes na maioria dos estudos analisados. Os achados ressaltam a importância da identificação precoce e gestão cuidadosa desses fatores de risco para melhorar os resultados clínicos em pacientes críticos com COVID-19.

Esta revisão fornece evidências que podem guiar a prática dos profissionais de saúde, destacando os principais fatores de risco associados às complicações respiratórias e neurológicas em pacientes críticos com COVID-19. Os resultados oferecem subsídios valiosos para aprimorar a prevenção e o manejo dessas condições, contribuindo para a formulação e implementação de uma assistência mais eficaz.

# Translation: Prevalencia de complicaciones sistémicas de COVID-19 en pacientes críticos: revisión sistemática con metaanálisis ^*^

**DOI:** 10.1590/1518-8345.7568.4607

**Authors:** Namie Okino Sawada, Tiago Marques dos Reis, Patrícia Scotini Freitas, Camila Mendonça de Moraes, Bianca de Moura Peloso-Carvalho, Munyra Rocha Silva Assunção, Camila Maria Silva Paraizo Horvath, Ranile Santos Silva, Karina Dal Sasso Mendes

**Affiliations:** 1 Universidade Federal de Alfenas, Alfenas, MG, Brasil.; 2 Universidade de São Paulo, Escola de Enfermagem de Ribeirão Preto, Centro Colaborador de la OPS/OMS para el Desarrollo de la Investigación en Enfermería, Ribeirão Preto, SP, Brasil.; 3 Universidade Federal do Rio de Janeiro, Escola de Enfermagem, Macaé, RJ, Brasil.; 4 Becaria de la Coordenação de Aperfeiçoamento de Pessoal de Nível Superior (CAPES), Brasil.; 5 Becaria del Conselho Nacional de Desenvolvimento Científico e Tecnológico (CNPq), Brasil.; 6 Universidade José do Rosário Velano, Faculdade de Medicina, Alfenas, MG, Brasil.

**Keywords:** COVID-19, SARS-CoV-2, Comorbilidad, Enfermedad Crítica, Cuidados Críticos, Revisión Sistemática

## Abstract

identificar la prevalencia de las complicaciones sistémicas de la COVID-19 en pacientes críticos, para estimar las condiciones clínicas que pueden presentar peor pronóstico cuando se asocian a la COVID-19.

revisión sistemática con metaanálisis de estudios observacionales, basada en las recomendaciones del JBI *Manual for Evidence Synthesis* para revisiones sistemáticas de prevalencia e incidencia, en seis bases de datos y literatura gris, período 2020 a 2024, en los idiomas portugués, inglés y español, habiéndose identificado 2393 publicaciones. La selección, la extracción de datos y la evaluación metodológica de los estudios fueron realizadas por dos investigadores independientes. El metaanálisis se realizó mediante el método estadístico transformacional de Freeman Tukey de efecto aleatorio.

se incluyeron diez artículos. El metaanálisis de siete artículos que abordaban las complicaciones respiratorias por COVID-19 demostró una prevalencia media de 42%, (IC95% 0,2-0,66) con heterogeneidad I^2^=97,4; el metaanálisis de 4 artículos que involucraron la prevalencia de las complicaciones neurológicas por COVID-19 mostró una prevalencia media de 62%, (IC95% 0,49-0,74) con heterogeneidad I^2^=87,4 y el metaanálisis de 8 estudios mostró una prevalencia de la mortalidad por COVID-19 de 33%, (IC95% 0,23-0,44) con heterogeneidad I^2^=93,6.

las complicaciones respiratorias y neurológicas fueron las más prevalentes en los estudios revisados. Registro PROSPERO: CRD42020214617.

## Destacados

**(1)** Las complicaciones respiratorias y neurológicas se destacaron como las más prevalentes. **(2)** Los principales factores de riesgo son el sexo masculino, las comorbilidades, las enfermedades respiratorias y cardíacas. **(3)** La evidencia puede orientar la praxis de los profesionales de la salud. **(4)** La evidencia puede ayudar en la implementación de la asistencia a los pacientes críticos.

## Introducción

El coronavirus, perteneciente a la familia homónima y responsable del desarrollo de infecciones respiratorias, asoló el mundo tras la diseminación de una nueva cepa del SARS-CoV denominada SARS-CoV-2, que causa la enfermedad COVID-19. El 11 de marzo de 2020, la Organización Mundial de la Salud clasificó la distribución geográfica de la enfermedad como pandemia, situación que perduró por casi 30 meses^(1-3)^. A partir de entonces, se implementaron medidas de prevención como el distanciamiento social, la cuarentena y el uso de máscaras, con el objetivo de reducir la transmisibilidad y la letalidad de la enfermedad^(4)^. Estas medidas resultaron en sufrimiento psicológico y trastornos mentales, como ansiedad, depresión y estrés^(5)^. Además, se comenzaron a observar complicaciones sistémicas causadas por la enfermedad, principalmente mediante compromisos en los sistemas pulmonar y cardiovascular^(6-7)^.

El Centro de Control y Prevención de Enfermedades de los Estados Unidos de América (CDC) del 14 de octubre de 2021 mostró que la COVID-19 no afectó de manera equitativa a todos los grupos poblacionales^(8)^. El riesgo de complicaciones derivadas de esta enfermedad aumenta proporcionalmente al número de condiciones subyacentes que presenta una persona. Estudios previos indican que individuos con ciertas deficiencias tienen mayor probabilidad de contraerla y presentar desenlaces adversos^(9-10)^. Además, algunas condiciones crónicas, como enfermedades pulmonares, enfermedades cardíacas, diabetes y obesidad, son más prevalentes en determinadas poblaciones minoritarias, afectando a una franja etaria más joven. En los Estados Unidos, se observó un aumento estimado de 700.000 muertes en exceso, con el mayor incremento porcentual en la mortalidad entre adultos de 25 a 44 años y entre hispánicos o latinos^(8)^. En 2022, la COVID-19 fue la cuarta causa principal de muerte en ese país^(11)^.

Evidencias respaldadas por revisiones sistemáticas destacan que condiciones subyacentes que aumentan el riesgo de progresión a enfermedad grave debido a la COVID-19 incluyen enfermedades pulmonares^(12-16)^, cáncer^(17-18)^, enfermedad cerebrovascular^(18-21)^, enfermedad renal crónica^(22-23)^, enfermedad hepática crónica (cirrosis, enfermedad hepática grasa no alcohólica, enfermedad hepática alcohólica, hepatitis autoinmune)^(24-26)^, Diabetes Mellitus (DM), Diabetes Mellitus Tipo 1 (DM1) y Diabetes Mellitus Tipo 2 (DM2)^(27)^, problemas cardíacos (como insuficiencia cardíaca, enfermedad arterial coronaria o miocardiopatías)^(28-29)^, tabaquismo actual y pasado^(30-32)^, tuberculosis^(33-34)^, obesidad^(35)^, embarazo y embarazo reciente^(36)^ y trastornos de salud mental (trastornos del ánimo, incluida la depresión y los trastornos del espectro de la esquizofrenia)^(37-38)^.

Ante lo expuesto, los grupos de riesgo parecen tener una mayor probabilidad de desarrollar complicaciones sistémicas por COVID-19. Se consideró como pacientes críticos a aquellos gravemente enfermos, con inestabilidad clínica o riesgo de inestabilidad del sistema vital con riesgo de muerte y, de esta forma, ingresados en la unidad de cuidados intensivos (UCI)^(39)^. Así, este estudio tuvo como objetivo identificar la prevalencia de las complicaciones sistémicas de la COVID-19 en pacientes críticos. Se espera que el estudio pueda contribuir a estimar las condiciones clínicas de los pacientes que pueden presentar un peor pronóstico cuando se asocian a la COVID-19.

## Método

### Tipo de estudio

Se trata de una revisión sistemática que siguió las directrices del *Centro Brasileiro de Pesquisas Baseadas em Evidências* COBE-UFSC^(40)^, y el *checklist Preferred Reporting Items for Systematic Reviews and Meta-Analyses* (PRISMA)^(41)^. Para su desarrollo se organizaron seis etapas: (1) elaboración de la pregunta de investigación; (2) preparación de la estrategia de búsqueda preliminar; (3) elección de las bases de datos; (4) definición de los criterios de elegibilidad; (5) recolección de datos y análisis de los riesgos de sesgo y (6) relato de la revisión^(40)^. El protocolo de la revisión sistemática fue registrado en el *International Prospective Register of Ongoing Systematic Reviews* – PROSPERO con el número CRD42020214617.

### Pregunta de estudio

Se buscó, con esta revisión, responder a la siguiente pregunta: ¿Cuál es la prevalencia de las complicaciones sistémicas resultantes de la contaminación por SARS-CoV-2 en adultos con COVID-19 en estado crítico? Para formular esta pregunta, se utilizó la estrategia PECOS^(40)^, siendo: P- Participantes: adultos con COVID-19; E- Exposición: contaminación por COVID-19; C- Control: ausencia de contaminación; O- Desenlace: complicaciones sistémicas; S- estudios observacionales.

### Período de recolección de datos

La realización de esta revisión sistemática comprendió el período del 30 de agosto de 2020 al 30 de marzo de 2024.

### Criterios de elegibilidad

Se incluyeron estudios observacionales que abordaron adultos con COVID-19 en condición crítica, considerando aspectos epidemiológicos, manifestaciones clínicas, diagnóstico, prevención y control de la enfermedad. Como criterio de exclusión, se desestimaron estudios de revisión, estudios de caso-control, relatos de casos, estudios y series de casos, publicaciones de resúmenes de anales de eventos científicos y editoriales.

### Recolección de datos y estrategia de búsqueda

Las bases de datos utilizadas fueron MEDLINE (a través de *PubMed*), EMBASE, LILACS, *Web of Science Core Collection*, *Scopus* y CINAHL. Para la estrategia de búsqueda, se emplearon los descriptores del *Medical Subject Headings* (MeSH), Emtree, Títulos CINAHL y los Descriptores en Ciencias de la Salud (DeCS/MeSH), además de los términos sinónimos, considerando las especificidades de cada base de datos. Como ejemplo, se destaca la estrategia de búsqueda implementada en PubMed: ((“COVID-19”[Supplementary Concept] OR “COVID-19”[All Fields] OR “2019 novel coronavirus disease”[All Fields] OR “COVID19”[All Fields] OR “COVID-19 pandemic”[All Fields] OR “SARS-CoV-2 infection$”[All Fields] OR “COVID-19 virus disease”[All Fields] OR “2019 novel coronavirus infection$”[All Fields] OR “2019-nCoV infection$”[All Fields] OR “coronavirus disease 2019”[All Fields] OR “coronavirus disease-19”[All Fields] OR “2019-nCoV disease”[All Fields] OR “COVID-19 virus infection$”[All Fields] OR “Coronavirus Infections”[Mesh] OR “Coronavirus Infections”[All Fields] OR “Coronavirus Infection$”[All Fields] OR “Middle East Respiratory Syndrome”[All Fields] OR “MERS (Middle East Respiratory Syndrome)”[All Fields]) AND (“Systemic complications”[Title/Abstract] OR “Systemic complication$”[All Fields] OR “Covid-19 complication$”[All Fields] OR “Covid 19 complication$”[All Fields] OR “Covid19 complication$”[All Fields] OR “SARS-CoV-2 complication$”[All Fields])). La búsqueda se realizó el 30 de agosto de 2023.

Además, se investigó también la literatura gris en las fuentes *Google Scholar*, *Proquest* y *OpenGrey*, y se efectuó una búsqueda manual en la lista de referencias de los artículos incluidos. Los resultados identificados en cada base de datos fueron exportados a *EndNote* 20 versión *desktop* para la eliminación de duplicados. Posteriormente, los registros fueron exportados al *software online Rayyan* ^(42)^, para la selección de los estudios de forma individualizada por dos revisores, mediante la lectura de títulos y resúmenes, seguida de la selección del texto en su totalidad. Tras cada una de estas fases de selección, se realizó una reunión de consenso para discusión y alineación de los conflictos.

### Recolección de datos

La extracción de los datos se llevó a cabo utilizando el instrumento de la colaboración *Joanna Briggs Institute* (JBI)^(43)^ para estudios observacionales, abarcando las siguientes variables: país, contexto y lugar, características de los participantes, grupos, medidas de resultados y descripción de los principales resultados. La recolección fue realizada por dos investigadores de forma independiente y los conflictos fueron resueltos con un tercer revisor.

### Análisis de los datos

Se realizó una síntesis cualitativa considerando variables dependientes (tipo de complicación sistémica) e independientes (sexo, rango etario, presencia de comorbilidades y hallazgos de laboratorio). En la síntesis cuantitativa, se procedió a un metaanálisis de proporción utilizando el método estadístico de Freeman Tukey transformacional de efecto aleatorio, con un intervalo de confianza del 95%. El metaanálisis se realizó mediante el *software* de la colaboración JBI^(43)^, agrupando los estudios en cada grupo de acuerdo con prevalencias similares en relación con las complicaciones sistémicas de la COVID-19. Los análisis involucraron todos los artículos incluidos, independientemente de la calidad metodológica. Las complicaciones sistémicas de la COVID-19 se clasificaron según su etiología en neurológicas, respiratorias o muerte.

Para evaluar la calidad metodológica se utilizó la herramienta propuesta por la colaboración JBI^(44)^ para estudios de cohorte, que abarca once cuestiones: ¿Los dos grupos eran similares y reclutados en la misma población? ¿Se midieron las exposiciones de forma similar para asignar a las personas a los grupos expuestos y no expuestos? ¿Se midió la exposición de forma válida y confiable? ¿Se identificaron factores de confusión? ¿Se declararon estrategias para abordar los factores de confusión? ¿Los grupos/participantes estaban libres del desenlace al inicio del estudio (o en el momento de la exposición)? ¿Se midieron los resultados de forma válida y confiable? ¿Se reportó el tiempo de seguimiento y fue suficiente para que los resultados ocurrieran? ¿El seguimiento fue completo y, en caso contrario, se describieron y exploraron los motivos de la pérdida del seguimiento? ¿Se utilizaron estrategias para abordar el seguimiento incompleto? ¿Se utilizó un análisis estadístico adecuado? Esta evaluación fue realizada por dos revisores de forma independiente, y las dudas se discutieron en reuniones para buscar consenso. Para la interpretación del análisis se consideró la calidad metodológica general de cada estudio, la cual se clasifica como “Alta”, “Moderada” o “Baja”, a partir del conteo o porcentaje de respuestas “Sí” a los ítems de la herramienta. De esta forma, se adoptaron los siguientes puntos de corte: hasta 25% de respuestas afirmativas “Alto riesgo de sesgo”; de 25 a ≤75% “riesgo moderado de sesgo” y mayor al 75% “Bajo riesgo de sesgo”^(45)^.

La certeza de la evidencia no se realizó, dado que actualmente no existe una orientación formal del *Grading of Recommendations Assessment*, *Development and Evaluation* (GRADE) para revisiones sistemáticas de prevalencia e incidencia^(46)^.

## Resultados

La búsqueda identificó 2393 registros, siendo 97 de *PubMed*, 417 de *Web of Science Core Collection*, 556 de LILACS, 450 de *Scopus*, 501 de EMBASE, 26 de CINAHL, 100 de *Google Scholar* y 246 de *ProQuest*, y ninguno de *OpenGrey*. Tras la eliminación de duplicados y considerando los criterios de elegibilidad, se obtuvieron 10 estudios de cohorte. La Figura 1 muestra el diagrama de flujo del proceso de selección de los estudios incluidos en la revisión sistemática.

**Figura 1 -:** Diagrama de flujo de selección de los estudios incluidos en la revisión sistemática adaptado del *Preferred Reporting Items for Systematic Reviews and Meta-Analyses* (PRISMA) (n = 10), 2020-2024

Fuente: Adaptado del PRISMA^(41)^

La síntesis de los 10 estudios se describe en el Apéndice 1 y detalla la identificación del estudio, país de realización, contexto, características de los participantes, grupos, medidas de resultados y principales resultados y se puede consultar en https://doi.org/10.48331/scielodata.4BEZDJ.

Las proporciones de ocurrencias de las complicaciones sistémicas de la COVID-19 se describen en las Figuras 2, 3 y 4.

La Figura 2 muestra el metaanálisis de siete artículos que trataban de las complicaciones respiratorias por COVID-19. Se observó una prevalencia media (meta-prevalencia) de 42%, (IC95% 0,2-0,66) con heterogeneidad I^2^=97,4.

**Figura 2 -:** Metaanálisis de 7 artículos que trataban la prevalencia de complicaciones respiratorias por COVID-19, 2020-2024

La Figura 3 muestra el metaanálisis de 4 artículos que involucraron la prevalencia de las complicaciones neurológicas por COVID-19. Se observa una prevalencia media (meta-prevalencia) de 62%, (IC95% 0,49-0,74) con heterogeneidad I^2^=87,4.

**Figura 3 -:** Metaanálisis de 4 estudios que mostraron la prevalencia de complicaciones neurológicas por COVID-19, 2020-2024

La Figura 4 evidencia el metaanálisis de ocho estudios que mostraron la prevalencia de la mortalidad por COVID-19. Se puede observar una prevalencia media (meta-prevalencia) de 33%, (IC95% 0,23-0,44) con heterogeneidad I^2^=93,6.

**Figura 4 -:** Metaanálisis de ocho estudios que mostraron la prevalencia de la mortalidad por COVID-19, 2020-2024

La heterogeneidad se evaluó mediante la inconsistencia (I²) que describe la variación porcentual atribuida a ellos más allá del azar. Los puntos de corte considerados fueron: 25% baja; 50% moderada y 75% alta heterogeneidad, con valor de p<0,05^(47)^.

Con respecto a la evaluación de la calidad metodológica de los estudios de cohorte incluidos, el 10% presentó calidad metodológica alta y el 90% calidad moderada, es decir, el 10% de los estudios tienen bajo riesgo de sesgo y el 90% riesgo moderado de sesgo (Figura 5).

**Figura 5 -:** Evaluación de la calidad metodológica de los estudios de cohorte incluidos (n = 10), 2020-2024

## Discusión

Esta revisión sistemática tuvo como pregunta cuál es la prevalencia de las complicaciones sistémicas de la COVID-19 en pacientes críticos y encontramos como respuesta que las complicaciones respiratorias y neurológicas fueron las más prevalentes en los estudios revisados, indicando que los pacientes críticos con COVID-19 pueden presentar mayor riesgo de desarrollar estas complicaciones.

De los 10 estudios incluidos en esta revisión sistemática, tres se produjeron en Europa^(48-50)^, tres en Asia^(51-53)^, uno en América Central^(54)^, uno en América del Norte^(55)^ y dos en América del Sur^(56-57)^, caracterizando la COVID-19 como una pandemia con reflejos de gravedad en varios países y regiones del mundo.

Las publicaciones más antiguas datan de 2020, año del inicio de la emergencia sanitaria^(58)^. El número de participantes de los estudios de cohorte incluidos totalizó 1143 personas, con una media de 62 años, predominantemente del sexo masculino. En la mayoría de los estudios, los pacientes presentaron al menos una comorbilidad, siendo las más prevalentes la hipertensión arterial, Diabetes Mellitus, obesidad y enfermedades cardíacas.

En cuanto a las manifestaciones pulmonares en la COVID-19, estas pueden manifestarse de forma leve, moderada o grave. En la forma leve, pueden estar presentes síntomas como dolores en el cuerpo, tos o fiebre leve. En la forma moderada, la enfermedad puede presentarse como una neumonía leve, asociada a otros síntomas. Ya la forma grave de la enfermedad puede caracterizarse por una neumonía grave e hipoxia. En casos críticos que presentan hipoxia significativa y fallo de órganos, puede ser necesaria la hospitalización en la Unidad de Cuidados Intensivos (UCI) con soporte ventilatorio mecánico^(52,59-60)^.

Los estudios identificados en esta revisión sistemática revelaron que la ventilación mecánica se inició con mayor frecuencia en pacientes con enfermedades graves en comparación con aquellos con enfermedades no graves (ventilación no invasiva, 32,4% vs 0%; ventilación invasiva, 14,5% vs 0%)^(51)^. Un estudio^(56)^ que involucró 168 pacientes gravemente enfermos con COVID-19 (media de edad de 65 años, 66% del sexo masculino), indicó que la hipertensión arterial fue la comorbilidad más frecuente, afectando al 52,4% de los pacientes. De estos, el 67,9% requirió Ventilación Mecánica Invasiva (VMI), y ninguno fue tratado con ventilación no invasiva. La mayoría de los pacientes bajo VMI (73,7%) necesitó bloqueo neuromuscular debido a la hipoxemia grave y el 36% fue ventilado en posición prona. La permanencia media en la UCI fue de 13 días, con una tasa de mortalidad del 25%. El estudio evidenció que la ventilación mecánica estaba asociada con las mayores probabilidades de desarrollar *delirium* (OR: 42,1; IC95%: 13,0-137,1), con una mortalidad del 26,4% en los pacientes con *delirium* en comparación con el 15,8% en los pacientes sin dicho trastorno^(55)^.

Entre 710 pacientes con neumonía por SARS-CoV-2, se incluyeron 52 adultos gravemente enfermos. La media de edad fue de 59,7 años, siendo el 67% hombres y el 40% con enfermedades crónicas. La mayoría de los pacientes (61,5%) falleció a los 28 días, con una duración mediana de 7 días desde la admisión en la UCI hasta la muerte para los no sobrevivientes. En comparación con los sobrevivientes, los no sobrevivientes eran de mayor edad, tenían mayor probabilidad de desarrollar Síndrome Respiratorio Agudo [26 (81%) pacientes vs 9 (45%) pacientes] y mayor probabilidad de recibir ventilación mecánica [30 (94%) pacientes vs 7 (35%) pacientes], ya fuera invasiva o no invasiva. La mayoría de los pacientes presentaba disfunción orgánica, incluyendo Síndrome de Distrés Respiratorio Agudo (SDRA) (67%), lesión renal aguda (29%), enfermedad cardíaca (23%), disfunción hepática (29%) y neumotórax (2%). Treinta y siete (71%) requirieron VMI, y siete (13,5%) desarrollaron infección hospitalaria^(52)^.

Esta asociación también fue evidenciada en un estudio^(57)^, en el que los casos más graves de COVID-19 ocurren en pacientes ancianos, hipertensos y obesos, con un aumento significativo de la mortalidad cuando se asocia a la enfermedad renal.

Estudio^(54)^ reveló que el 30% de los pacientes requirieron VMI, siendo todos de edad igual o superior a 65 años y predominantemente del sexo femenino. De estos, el 83,3% fallecieron^(5)^, y cuatro presentaron SDRA grave o moderado (RR 16,7) de acuerdo con la clasificación de Berlín. La media de presión positiva espiratoria final ideal (PEEP) utilizada fue de 16,5 (DE=2,1) cmH_2_O (IC 15,6-17,4), con la modalidad ventilatoria inicial siendo ventilación controlada por volumen (VCV). En uno de los casos se adoptó la posición en decúbito prono después de las primeras 12 horas de admisión en la UCI. La posición prono fue contraindicada en los demás casos debido a la inestabilidad hemodinámica. El paciente que obtuvo éxito en el destete ventilatorio utilizó el modo Bi Presión positiva (BiPAP), lo que resultó en menores necesidades de sedación y relajación muscular, además de un tiempo menor de VMI (5 días), a pesar de la edad avanzada. No se empleó ventilación no invasiva.

El metaanálisis de prevalencia de las complicaciones respiratorias, resultando en la necesidad de VMI, fue del 42% (IC: 0,39-0,46, I^2^: 98) en una población total de 704 pacientes internados en UCI. Una revisión sistemática^(61)^ cuyo objetivo fue determinar si las presentaciones y manifestaciones de la COVID-19 se limitaban exclusivamente a las manifestaciones pulmonares, identificó una incidencia del 33% de Síndrome Respiratorio Agudo.

En cuanto a las complicaciones neurológicas, las evidencias provenientes de una revisión sistemática^(62)^ indicaron que las manifestaciones neurológicas más comunes asociadas a la COVID-19 incluyeron mialgia, cefalea, alteración sensorial, hiposmia e hipogeusia. Además, se observaron manifestaciones poco frecuentes del sistema nervioso central, como accidente cerebrovascular isquémico, hemorragia intracerebral, encefalomielitis y mielitis aguda, así como manifestaciones nerviosas periféricas, como síndrome de Guillain-Barré y parálisis de Bell, y manifestaciones musculares esqueléticas, como rabdomiólisis. Informes recientes sugieren que el daño hipóxico-isquémico puede ser la principal causa de los síntomas neurológicos en pacientes con COVID-19^(63)^.

Los estudios incluidos en esta revisión sistemática identificaron el *delirium* como la complicación neurológica más común. En uno de los estudios^(48)^ no se observó asociación entre el *delirium* y la duración de la ventilación mecánica. Por otro lado, otro estudio^(56)^ indicó una menor incidencia de este agravio en pacientes no sometidos a ventilación mecánica (23, 42,5%) en comparación con los pacientes en ventilación mecánica (61, 89,7%). Una investigación^(55)^ reveló que el *delirium* ocurrió en el 73,6% de los pacientes y que la ventilación mecánica estuvo asociada a las mayores probabilidades de desarrollar *delirium* o coma en el 76,4% (110/144). El 63% de los pacientes fueron positivos para *delirium* (OR: 42,1, IC95%: 13,0-137,1).

Una investigación^(53)^ demostró que, entre 52 pacientes en la UCI con COVID-19, la media de edad fue de 73 años, siendo que 19 (36,5%) tenían comorbilidades neurológicas preexistentes. Las complicaciones neurológicas de inicio reciente ocurrieron en 23 (44,2%) pacientes durante la hospitalización en la UCI.

El metaanálisis de la prevalencia de complicaciones neurológicas, que involucró a 458 pacientes internados en la UCI, reveló una prevalencia elevada del 62% (IC: 0,49-0,74, I2: 87,4).

La revisión sistemática con metaanálisis^(64)^ evidenció los factores de riesgo para mortalidad en la COVID-19, incluyendo un total de 14 estudios con 29.909 pacientes infectados por el virus, registrando 1.445 casos de muerte. El estudio reveló asociaciones significativas entre edad avanzada (≥65 vs <65 años) (OR agrupadas = 4,59, IC95% = 2,61–8,04, *p* < 0,001), sexo masculino en comparación con femenino (OR agrupadas = 1,50, IC95% = 1,06–2,12, *p* = 0,021) y el riesgo de muerte por infección de COVID-19.

Adicionalmente, condiciones como hipertensión (OR agrupada = 2,70, IC95% = 1,40–5,24, *p* = 0,003), enfermedades cardiovasculares (ECV) (OR agrupada = 3,72, IC95% = 1,77–7,83, *p* = 0,001), diabetes (OR agrupadas = 2,41, IC95% = 1,05–5,51, *p* = 0,037), enfermedad pulmonar obstructiva crónica (EPOC) (OR agrupadas = 3,53, IC95% = 1,79–6,96, *p* < 0,001) y cáncer (OR agrupados = 3,04, IC95% = 1,80–5,14, *p* < 0,001) se mostraron asociados a un mayor riesgo de mortalidad^(64)^.

Una investigación^(49)^ demostró un porcentaje mayor de complicaciones durante la hospitalización de pacientes con neumonía neumocócica bacteriemica. En contraste, los pacientes con neumonía por SARS-CoV-2 presentaron una mayor tasa de mortalidad hospitalaria (10,8% vs 6,8%; P = 0,004) y un mayor tiempo de hospitalización. En pacientes con neumonía por SARS-CoV-2, los predictores de mortalidad hospitalaria fueron: edad ≥65 años (OR 4,777, IC95% 2,814–8,111; P < 0,001); enfermedad cardíaca (OR 1,587, IC95% 1,007–2,341; P = 0,02); enfermedad hepática (OR 2,365, IC95% 1,199–4,662); estado mental alterado (OR 2,464, IC95% 1,586–3,827; P < 0,001); frecuencia respiratoria ≥30 respiraciones/min (OR 2,117, IC95% 1,362–3,289; P < 0,001); hipoxemia (OR 1,599, IC95% 1,045–2,447; P = 0,03); nitrógeno ureico en sangre ≥30 mg/dL (OR 1,864, IC95% 1,236–2,81; P = 0,003); neutrofilia (cada aumento de 10.000 unidades en el recuento de neutrófilos/μL) (OR 1,052, IC95% 1,018–1,086; P = 0,002); compromiso pulmonar bilateral (OR 1,868, IC95% 1,142–3,056; P = 0,013); derrame pleural (OR 2,894, IC95% 1,282–6,536; P = 0,011) y shock séptico (OR 2,781, IC95% 1,703–4,542; P < 0,001). Siete días o más de síntomas en la admisión fueron factores de protección contra la mortalidad (OR 0,464, IC95% 0,314–0,685; P < 0,001).

Los resultados de esta revisión sistemática corroboran el análisis del estudio^(64)^, ya que todos los incluidos destacaron factores de riesgo, con predominio del sexo masculino. En siete (70%) de las investigaciones, los pacientes presentaron al menos una comorbilidad, con predominio de hipertensión, diabetes, obesidad, enfermedades cardíacas y pulmonares. El metaanálisis de prevalencia de mortalidad en esta revisión sistemática para COVID-19 fue del 33% (IC: 0,23-0,44, I²: 93,6), involucrando una población de 2215 pacientes internados en UCI.

El estudio^(50)^ que evaluó la incidencia de complicaciones trombóticas y la mortalidad general en pacientes con COVID-19 internados en UCI durante la primera y segunda ola de la pandemia, reveló que, de los 358 pacientes internados en UCI, 144 pacientes fallecieron (15%). La incidencia acumulada ajustada de todas las complicaciones trombóticas después de 10, 20 y 30 días fue del 12% (intervalo de confianza del 95%: 9,8–15%), 16% (13–19%) y 21% (17–25%), respectivamente. Las características de los pacientes entre la primera y la segunda ola fueron comparables. La tasa de riesgo ajustada (HR) para mortalidad general en la segunda ola frente a la primera fue de 0,53 (IC95%: 0,41–0,70). El HR ajustado para cualquier complicación trombótica en la segunda versus la primera ola fue de 0,89 (IC95%: 0,65–1,2). En conclusión, se observó una reducción del 47% en la mortalidad en la segunda ola, pero la tasa de complicaciones trombóticas se mantuvo elevada y comparable a la de la primera ola. Se resalta la necesidad de cuidados cautelosos y de garantizar una tromboprofilaxis adecuada.

Otro aspecto importante es la admisión del paciente en la unidad de emergencia. Un estudio^(65)^ identificó la importancia del uso de la clasificación de riesgo en el momento de la admisión, en el que los pacientes con COVID-19 que obtuvieron puntuaciones de alerta temprana modificadas mayores que 4 estuvieron asociados a las categorías urgente, muy urgente y emergencial, y tuvieron mayor deterioro clínico, como insuficiencia respiratoria y shock, evolucionando en mayor medida hacia el fallecimiento. Esto demuestra que el protocolo de riesgo priorizó correctamente a los pacientes con riesgo vital.

En cuanto a las limitaciones del estudio, esta revisión sistemática no incluyó textos en idioma mandarín, ya que en el equipo no se contaba con investigadores que dominen ese idioma. Otra limitación fue la alta heterogeneidad de los estudios encontrados en el metaanálisis, la cual se atribuye al conjunto de complicaciones analizadas por los artículos. Quizás una laguna en el conocimiento sería la realización de estudios de cohorte con un solo tipo de complicación. Además, la mayoría de los estudios presentaron riesgo moderado de sesgo; solo uno mostró bajo riesgo de sesgo, por lo que existe una carencia de estudios con métodos más rigurosos.

Esta revisión sistemática proporciona una contribución significativa para el avance del conocimiento sobre las complicaciones sistémicas en pacientes internados en UCI. Al presentar evidencias detalladas sobre la prevalencia de complicaciones respiratorias, neurológicas y mortalidad, junto con la identificación de sus factores de riesgo, la revisión sirve como un instrumento valioso para orientar a los profesionales de la salud en el planeamiento y la prestación de asistencia a esta población crítica. La información reunida en este estudio tiene el potencial de mejorar las prácticas clínicas, promoviendo un enfoque más informado y eficaz en el cuidado de los pacientes con COVID-19 en estado crítico.

## Conclusión

Las complicaciones respiratorias y neurológicas se destacaron como las más prevalentes entre los pacientes críticos afectados por la COVID-19 en los estudios revisados. Los factores de riesgo significativos para complicaciones y mortalidad relacionadas con la COVID-19, tales como el sexo masculino y las comorbilidades de hipertensión, diabetes mellitus, obesidad, además de las enfermedades respiratorias y cardíacas, estuvieron consistentemente presentes en la mayoría de los estudios analizados. Estos hallazgos resaltan la importancia de la identificación temprana y la gestión cuidadosa de estos factores de riesgo para mejorar los resultados clínicos en pacientes críticos con COVID-19.

Esta revisión proporciona evidencias que pueden guiar la práctica de los profesionales de la salud, destacando los principales factores de riesgo asociados a las complicaciones respiratorias y neurológicas en pacientes críticos con COVID-19. Los resultados ofrecen insumos valiosos para optimizar la prevención y el manejo de estas condiciones, contribuyendo a la formulación e implementación de una asistencia más eficaz.
